# The forgotten joint score-12 is a valid and responsive outcome tool for measuring success following hip arthroscopy for femoroacetabular impingement syndrome

**DOI:** 10.1007/s00167-020-06138-7

**Published:** 2020-07-20

**Authors:** Patrick G. Robinson, C. S. Rankin, I. R. Murray, J. F. Maempel, P. Gaston, D. F. Hamilton

**Affiliations:** 1 Trauma and Orthopaedic Department, University of Edinburgh, 51 Little France Crescent, Edinburgh, EH16 4SA UK; 2 Department of Trauma and Orthopaedics, Royal Prince Alfred Hospital, Camperdown, NSW Australia

**Keywords:** Hip, Psychometric, FJS-12, Ceiling, FAI

## Abstract

**Purpose:**

The forgotten joint score-12 (FJS-12) is an outcome questionnaire designed to evaluate joint awareness. The responsiveness and validity of the English language version of the FJS-12 in patients undergoing hip arthroscopy for femoroacetabular impingement (FAI) is not known.

**Methods:**

Consecutive patients undergoing hip arthroscopy for a diagnosis of FAI were prospectively followed up over a 1 year period. Patients completed preoperative and postoperative FJS-12, EuroQol 5 Dimension (EQ-5D-5L), and the 12-item international hip outcome tool (iHOT-12). We evaluated construct validity with Spearman correlation coefficients for the FJS-12, and responsiveness by way of effect size and ceiling effects.

**Results:**

Forty-six patients underwent hip arthroscopy, of which 42 (91%) completed post-operative PROMs at 1 year follow-up. Construct validity was strong with the iHOT-12 (*r *= 0.87) and also the EQ-5D-5L (*r *= 0.83). The median postoperative FJS score was 50.2 (IQR 64). The mean change in score for the FJS-12 was 31 points (SD 31) (*p* < 0.001), with an effect size (Cohen’s *d*) of 1.16. Preoperatively, three patients scored the lowest possible value resulting in a floor effect of 7.1%. Similarly, only three patients (7.1%) scored the best possible score post-operatively.

**Conclusion:**

This is the first evaluation of the joint awareness concept in the English language version of the FJS-12 following hip arthroscopy for FAI. The FJS-12 is a valid and responsive tool for the assessment of this cohort of patients.

**Level of evidence:**

II.

**Electronic supplementary material:**

The online version of this article (10.1007/s00167-020-06138-7) contains supplementary material, which is available to authorized users.

## Introduction

Hip arthroscopy has been shown to be an effective treatment for the management of femoroacetabular impingement (FAI) [5, 15]. It is typically performed in a young, active population with the ideal outcome being a patient who is fully functional and without symptoms.

The success of such interventions needs to be assessed with appropriate tools that are valid and responsive to evaluate change following the intervention. The current patient reported outcome measures (PROMs) used to assess the results of hip arthroscopy are an improvement on previous generic health-related outcome measures or outcome measures designed for hip arthroplasty. Although they have been shown in some studies to have satisfactory responsiveness and validity, a recent review using Consensus-based Standards for the selection of health Measurement Instruments (COSMIN) guidelines could not recommend the use of the Hip Disability and Osteoarthritis Outcome Score (HOOS), Hip Sports Activity Score (HSAS), modified Harris Hip Score (mHHS), Non-Arthroplasty Hip Score (NAHS), the Super Simple Hip Score for Younger Patients (SUSHI), and Western Ontario and McMaster Universities Arthritis Index (WOMAC-12) based on current available psychometric analysis. Recent analysis of the responsiveness and ceiling effects of the 12 item international hip outcome tool (iHOT-12) found excellent responsiveness with a large effect size to postoperative change. However, there were concerns regarding the ceiling effect present for high performing groups, particularly young males [18].

The forgotten joint score (FJS-12) was designed to assess awareness of the joint during everyday life and activities and it has been shown to be an extremely useful tool in assessing high performing groups in arthroplasty [6, 11, 19]. More recently, it has been shown to be a valid and responsive tool in assessing patients following anterior cruciate ligament reconstruction [1] and first-time patellar dislocation [9]. With the strong responsiveness and validity shown by the FJS-12 in high functioning arthroplasty patients, it is possible that this outcome tool may also be effective in detecting differences in patient outcomes following hip arthroscopy.

Bramming et al. reported high relative reliability and responsiveness of the FJS-12 in hip arthroscopy using the Dutch translated version of the score in a Dutch population [2], but this had a comparatively short follow-up period of 6 months. A previous meta-analysis has shown substantial improvements following hip arthroscopy between 6 month and 1 year follow-up [8], and therefore, this study may not have captured the clinically relevant recovery period and improvement following surgery. The purpose of this study was to assess the responsiveness and validity of the English version of the FJS-12 and the concept of ‘joint awareness’ at longer follow-up (1 year) in patients who underwent hip arthroscopy for FAI. It was hypothesised that the FJS-12 would show good validity and responsiveness to change when used to assess patients following hip arthroscopy for a diagnosis of FAI.

## Materials and methods

A prospective cohort study was conducted. All patients undergoing hip arthroscopy over a 1-year period between January 2018 and January 2019 were included. Inclusion criteria were a diagnosis of FAI in patients of any gender or age who had failed non-operative treatment including analgesia and physiotherapy. Patients with Kellgren–Lawrence classification ≥ 2 were excluded from the analysis. A single surgeon performed all procedures. Included patients had been diagnosed by the treating surgeon with FAI (using clinical history, examination, plain radiographs, and magnetic resonance arthrogram where appropriate) and had previously exhausted conservative management. Joint injections were used to confirm the origin of symptoms in cases of doubt. Patients completed preoperative FJS-12, EuroQol 5D-5L (EQ-5D-5L), and iHOT-12 questionnaires 2 weeks prior to surgery at the pre-assessment clinic and again at 1 year postoperatively. Satisfaction data were also collected at 1 year postoperatively. Institutional review board approval was not sought for this study in keeping with advice from the local research ethics service [Scotland (16/SS/0026)].

### Surgical technique

The supine distractor was used for patient positioning. Image intensifier was used to confirm joint distraction. Superolateral and anterior portals were used to access the hip joint. These were expanded with sequential dilators to allow instrument access. The paralabral recess was opened and a high-speed burr was used to resect the pincer lesion of the acetabular rim and enable a flat surface for anchor placement. If the labrum was repairable, Stryker Cinchlock (Stryker, Mahwah, NJ, USA) anchors were used in a vertical mattress fashion with Cobraid sutures to repair the labrum. The traction was then released and attention was turned to the femoral head/neck junction. If a CAM lesion was identified, it was resected using a high-speed burr. Flexion was used to reach the anterior most aspects of the neck. An on-table impingement manoeuvre was performed to assess clearance of the femoral neck from the acetabulum under direct vision. Final orthogonal X-ray views were obtained to ensure adequate bony resection. The capsule was not repaired.

### Outcome measures

The FJS-12 contains 12 questions which are scored with a Likert scale ranging from 0 to 4. A lower score on the Likert scale equates to less awareness of the joint. The total sum score is converted into a scale ranging from 0 to 100, with higher scores reflecting less awareness of the joint during activities of daily living. The iHOT-12 was developed to assess the outcomes of hip arthroscopy. It contains 12 questions scored by rating answers on a visual analogue scale (VAS), with the mean value of all the questions equating to the total score. The total score ranges from 0 to 100 with a higher scoring reflecting less symptoms and better function. The EQ-5D-5L is made up of an index score and a visual analogue scale. The index consists of five domains which include mobility, self-care, usual activities, pain/discomfort, and anxiety/depression. Scores range from − 1 to + 1. The visual analogue scale (VAS), is a self-assessment of a patient’s health state, and is scored between 0 and 100. Patient satisfaction was assessed using a five point Likert scale. Patients were asked to report how satisfied they were with their operated hip on a scale of very satisfied, satisfied, neither satisfied nor dissatisfied, dissatisfied, and very dissatisfied.

### Statistical analysis

Statistical analysis was undertaken using Statistical Package for Social Sciences (SPSS) software (IBM, Inc., Armonk, New York, United States) v24. Normality was assessed using Kolmogorov–Smirnov testing. Continuous, normally distributed data were reported as mean with standard deviation and were compared using two-tailed Student’s *t* tests. Non-parametric data were reported as median with interquartile range and compared using the Wilcoxon signed-rank test for related samples and Mann–Whitney *U* test for unrelated samples. A *p* value of < 0.05 was considered statistically significant.

Construct validity was assessed by evaluating the convergence of the FJS-12 to the iHOT-12 and EQ-5D-5L using Spearman correlation coefficients. Values of 0.6–0.79 were considered to have a strong relationship and 0.9–1.0 as very strong [4]. Kruskal–Wallis testing was used to test for differences in outcomes (FJS-12, iHOT-12, and EQ-5D-5L) based on the level of satisfaction. The categories of ‘dissatisfied’ and ‘very dissatisfied’ were combined for the purposes of analysis due to low responses in both sections. A construct approach to responsiveness with hypothesis testing before and after intervention was used in line with COSMIN guidelines [12]. Ceiling effects are described as the number of patients obtaining the highest possible score on iHOT-12.

Change over time, from preoperative assessment to final follow-up following surgery (minimum 1 year), is presented as the effect size (Cohen’s *d*). A large effect is deemed to be *d* ≥ 0.8 [3]. Effect sizes were assessed for all patients and further evaluated within known subgroup comparisons of age, gender, and BMI.

## Results

Forty-six patients underwent hip arthroscopy at our institution in the period under review; 42 patients (91%) completed post-operative PROMs at 1 year follow-up. Patient characteristics are summarized in Table 1 and the specific surgical procedures performed are detailed in Table 2. The median postoperative FJS-12 score was 50.2 (IQR 64) and the median iHOT-12 score was 66 (IQR 40). The median postoperative EQ-5D-5L index score was 0.735 (IQR 0.304) and the median postoperative VAS score was 80 (IQR 32). Two patients complained of postoperative thigh paresthesia. One patient underwent repeat arthroscopy and revision of the labral repair secondary to a traumatic tear following a fall 11 months postoperatively, and one patient underwent a total hip replacement at 11 months for progression of symptoms and of radiographic osteoarthritis.

**Table 1:** Patient characteristics

	Responders (n = 42)
Age: mean (SD)	30 (8.4)
Range	17–45
Sex	
Male	14 (33.3%)
Female	28 (66.6%)
Side	
Left	21 (50%)
Right	21 (50%)
BMI: mean (SD)	25 (3.8)
Median preoperative iHOT-12 (IQR)	34.0 (21.3)
Median preoperative FJS-12 (IQR)	15.0 (23.0)

*SD* standard deviation, *IQR* interquartile range

**Table 2:** Summary of procedures performed

	*N* (%)
Acetabular procedures	
Labral repairs	
With or without rim recession	34 (38%)
With microfracture with or without rim recession	5 (6%)
Labral resection	
With or without rim recession	4 (4%)
With microfracture and rim recession	2 (2%)
Femoral procedure	
Cam removal	
Isolated cam removal	41 (46%)
With osteophyte removal	3 (3%)
With microfracture	1 (1%)

### Construct validity

To assess the construct validity of the FJS-12 was correlated (convergence validity) to the iHOT-12 and the EQ-5D-5L. Using Spearman’s rank correlation coefficient, there was a strong correlation between the FJS-12 and iHOT-12 (*r *= 0.87) (see supplementary 1) as well between the FJS-12 and the EQ-5D-5L (*r* = 0.83) (see supplementary 2). Similar findings were noted when comparing the iHOT-12 to the EQ-5D-5L (*r *= 0.86). One year postoperative scores of the FJS-12, iHOT-12 and EQ 5D index and their relationship to satisfaction are presented in Table 3.

**Table 3:** Comparison of median FJS, iHOT-12, and EQ 5D scores with satisfaction

	Very satisfied (*n* = 16)	Satisfied (*n* = 10)	Neither satisfied nor dissatisfied (*n* = 9)	Dissatisfied or very dissatisfied (*n* = 7)	*p* value
Median FJS-12 score	79.9 ± 21.4	43.6 ± 32.2	27.0 ± 21.7	9.7 ± 11	< 0.001
Median iHOT-12 score	83.9 ± 13.9	62.0 ± 18.6	36.7 ± 18.1	33.9 ± 28.6	< 0.001
Median EQ 5D Index	0.873 ± 0.134	0.660 ± 0.207	0.521 ± 0.200	0.388 ± 0.27	< 0.001

*FJS-12* Forgotten Joint Score, *iHOT-12* 12 item international hip outcome tool, *EQ* *5D* EuroQol 5D-5L, *SD* standard deviation

### Floor and ceiling effects

Preoperatively, three patients (7.1%) scored the lowest possible score in the FJS-12 and no patient scored the maximum score. Postoperatively, five patients (11.9%) scored the lowest possible score in the FJS-12 and three patients (7.1%) scored the best possible score. Eight patients (19.0%) scored within 10% of the maximum score.

### Responsiveness—sensitivity to changes

**Fig. 1:**
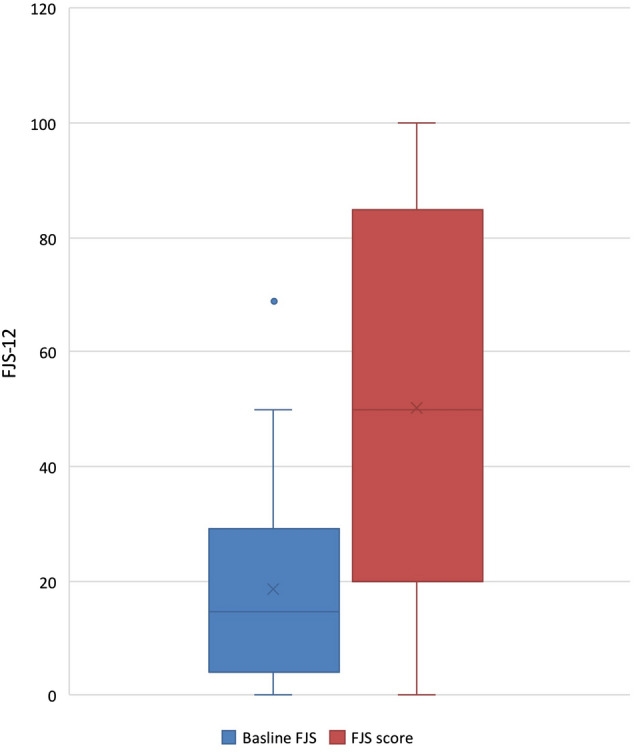
Change in FJS from baseline to 1 year follow-up

The mean change in score for the FJS-12 was 31 points (SD 31) (*p* < 0.001), with an effect size (Cohen’s *d*) of 1.16 (Fig. 1). We took the surrogate marker of one half of the standard deviation of the difference in pre- and postoperative outcome scores to estimate the minimally clinical important difference (MCID) in score for this population. We find an MCID estimate of 15.3 points.

### Subgroup analysis

**Fig. 2:**
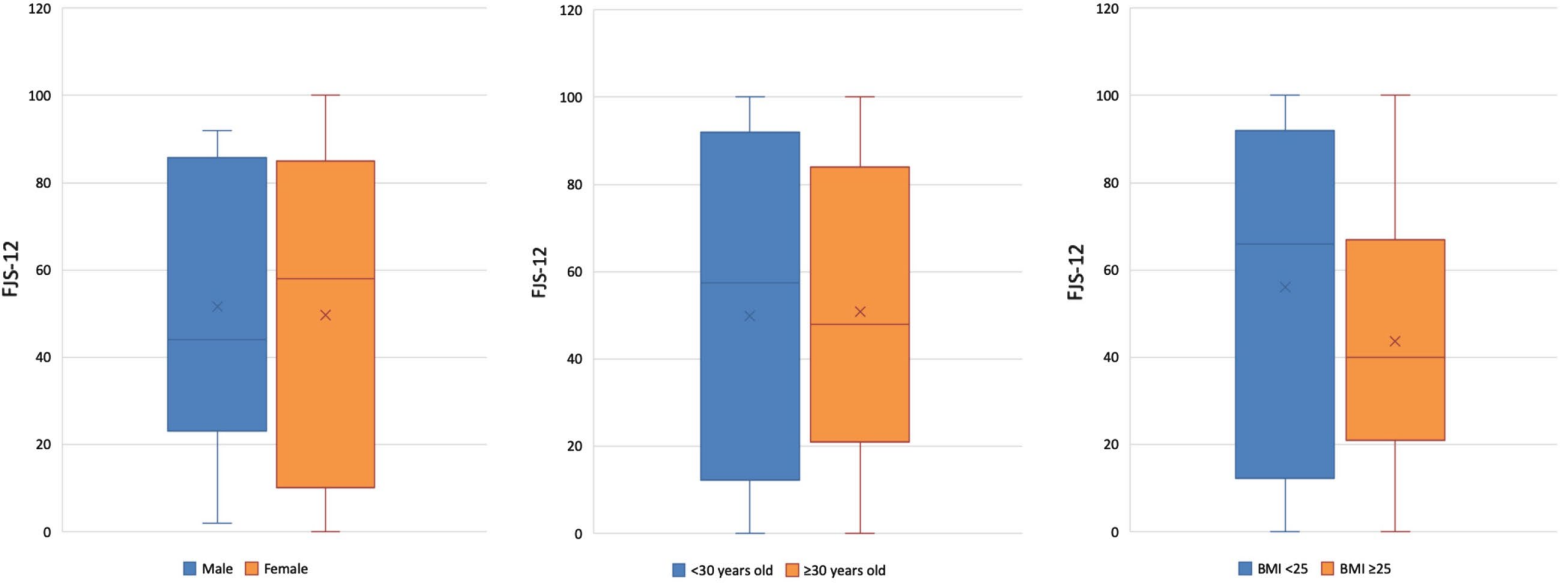
Postoperative FJS-12 scores by known group comparators: **a** Boxplot of females and males, **b** Boxplot of patients under 30 years old and equal to or over 30 years old. **c** Boxplot of patients with BMI under 25 and greater or equal to 25

The median FJS-12 in < 30 years old at 1 year follow-up was 57.5 (IQR 75.3) and 45.0 (IQR 35.5) for patient ≥30 years old (*p* = n.s.). The median FJS-12 in males at 1 year follow-up was 44.0 (IQR 61.0) and for females was 66.0 (IQR 75.0) (*p* = n.s.). The median FJS-12 in patients with BMI scores < 25 was 66.0 (IQR 75) and ≥25 was 40.0 (IQR 45) (*p* = 0.263) (Fig. 2).

## Discussion

The principle finding of this study was that the FJS-12 was seen to be a valid and responsive tool for measuring outcomes following hip arthroscopy for FAI. The FJS-12 showed strong correlation to the iHOT-12, which was designed and has been previously validated for this specific surgical population. The concept of joint awareness has been well explored and validated in arthroplasty populations; however, more recently, the score has been seen to have good measurement range when applied to younger, more active populations such as ACL reconstruction [1]; however, no previous study has investigated its use in patients undergoing non-arthroplasty hip surgery for FAI at greater than 6 month follow-up or using the English language version of the FJS-12.

Calculation of the effect size of the English language version of the FJS-12 shows that this outcome measure is very responsive to change. The patients’ outcomes were reviewed at a clinically relevant time point, 1 year following surgery. The only comparable data come from a Dutch language study in patients undergoing hip arthroscopy, where an effect size of 0.6 was reported at 6 month follow-up [2]. A substantially greater effect size of 1.16 was demonstrated at 1 year. This may suggest that patients continue to improve between 6 and 12 months. Clinically relevant improvements in sporting function following hip arthroscopy for FAI have previously been reported to begin between 6 and 12 months [8]. Therefore, reporting on psychometric qualities of the FJS-12 at 6 months but not beyond may give an incomplete picture and fail to report important information about subsequent changes in function that occur beyond this time point.

The minimal clinically important difference has not been reported for the FJS-12 in patients undergoing hip arthroscopy. Using half the standard deviation as a surrogate estimate [10, 13, 14], the MCID was calculated to be around 15 points. Pending formal evaluation, this is a useful guide to what is likely the MCID for FJS-12 in this population. The MCID of the FJS-12 in total knee arthroplasty has been reported as between 11 and 14 points [7], lending wider credibility to this estimate. This may help clinicians interpret FJS-12 scores reported in the context of hip arthroscopy for FAI.

7.1% of patients scored the best possible score, suggesting there to be minimal ceiling effect when using the FJS-12 in this population. However, 19% of patients scored within 10% of the maximum score, highlighting excellent outcomes in some patients. The distribution of post-operative scores (Fig. 1) highlights the wide range of outcomes achieved following FAI arthroscopy.

Interestingly, 11.9% of patients scored the worst possible score at 1 year follow-up. This would mean these patients reported being aware of their joint ‘most of the time’. It is important to qualify that joint awareness as a construct does not necessarily measure absolute functional ability or disability. Such patients may have been performing to a high level of function but aware of their joint while doing so. The FJS-12 offers an alternative tool to measuring clinical success following hip arthroscopy FAI. It is important to have a tool that has the measurement range to capture the physical changes and activity levels that the patient feels are important to their daily life. Hip arthroscopy is typically performed in young sporting populations. Although we were not powered to specifically evaluate subgroups, it may be that the FJS-12 is more suitable than the existing tools to capture changes in highly functioning patients, where ‘awareness’ of their joint during physical activities is an important factor for determining satisfaction and success. Large datasets, however, will have to be interrogated to robustly address this research question.

This study must be interpreted in light of its limitations. We report a comparatively modest number of patients for analysis; however, this represents the entire, consecutive throughput from a regional centre over the period of 1 year for this procedure, the sample size compares well to other studies in this field [2] and the key measure of responsiveness used in this study (effect size) is independent of sample size [17]. The iHOT-12 was used to evaluate the validity of the FJS-12 as a tool for assessing outcomes in hip arthroscopy. This tool was specifically designed to evaluate outcomes following hip arthroscopy; however, it has been recently demonstrated that it may suffer ceiling effects in better preforming subgroups of patients such as young males [16]. We were unable to demonstrate statistically significant differences in FJS-12 scores across subgroups for gender or BMI, despite relatively large differences in median FJS-12 score, suggesting that the study is underpowered for this type of subgroup analysis.

## Conclusion

The FJS-12 is a valid and responsive tool with which to assess the outcomes of patients with FAI undergoing hip arthroscopy. These findings conclude the FJS-12 questionnaire and more generally the construct of joint awareness is suitable for assessing outcomes in this young and more active population, in addition to the more commonly applied area of hip arthroplasty.

## Electronic supplementary material

Below is the link to the electronic supplementary material.

Supplementary file1 (tiff 4856 kb)

Supplementary file2 (tiff 4856 kb)

## Data Availability

The datasets used and/or analysed during the current study are available from the corresponding author on reasonable request.
