# Supplementary figures and images for: The forgotten joint score-12 is a valid and responsive outcome tool for measuring success following hip arthroscopy for femoroacetabular impingement syndrome
Source: Knee Surg Sports Traumatol Arthrosc. 2020 Jul 20;29(5):1378–84. doi: 10.1007/s00167-020-06138-7 (PMC8038977; doi:10.1007/s00167-020-06138-7)

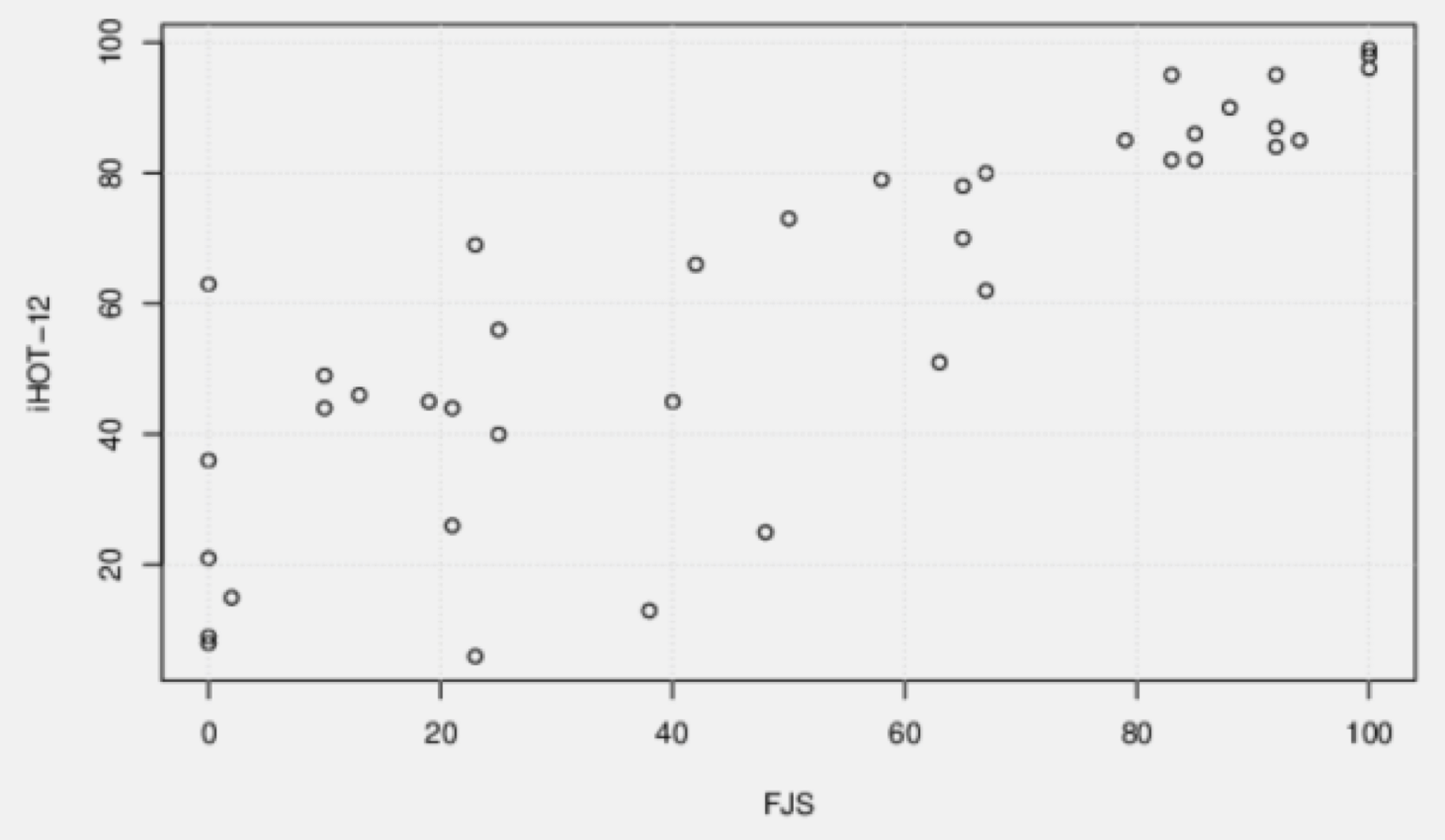

Supplement: Supplementary file 1 — Supplementary file1 (tiff 4856 kb) [file 167_2020_6138_MOESM1_ESM.tiff]

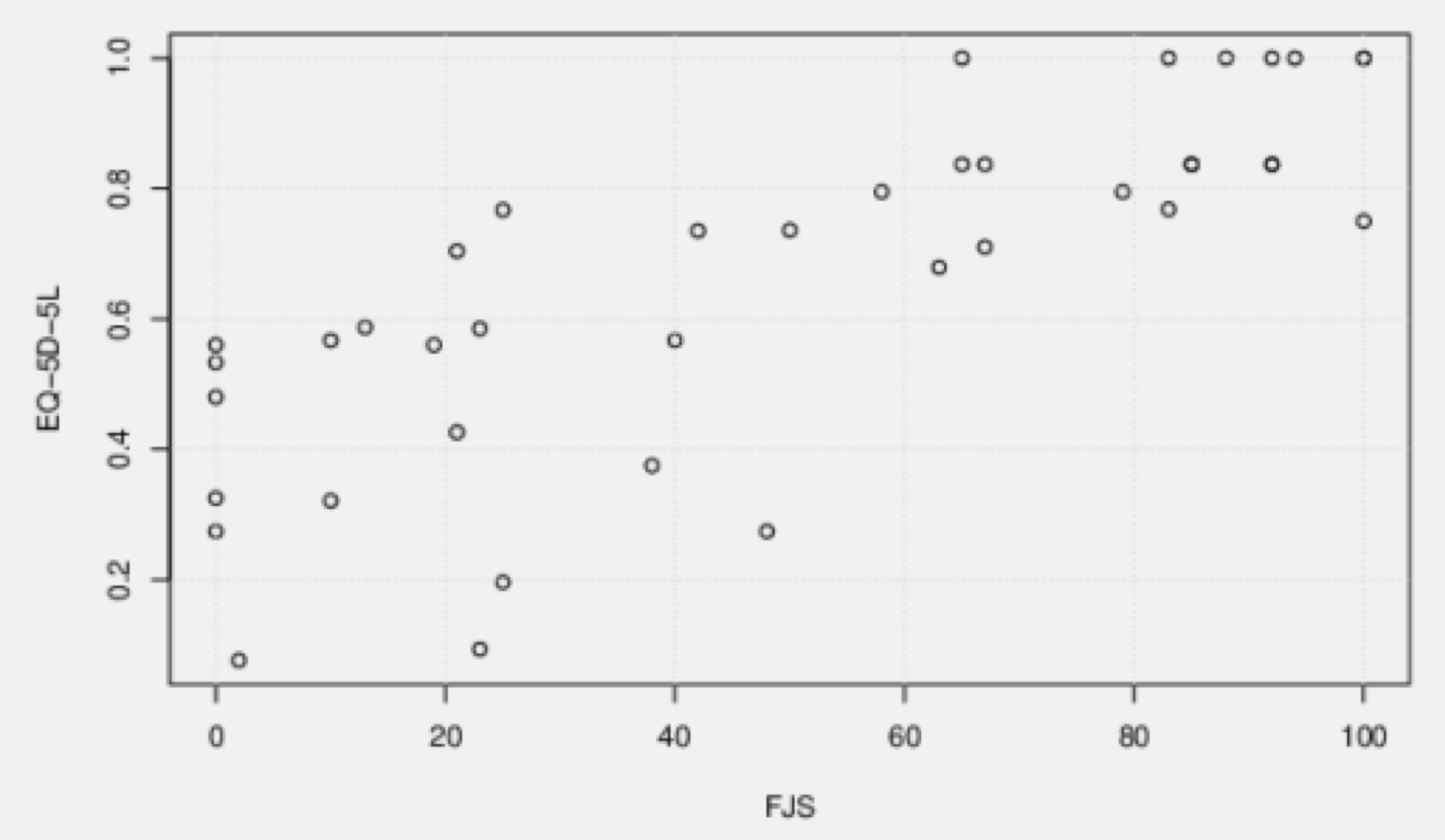

Supplement: Supplementary file 2 — Supplementary file2 (tiff 4856 kb) [file 167_2020_6138_MOESM2_ESM.tiff]
